# Supplementary material for: A Comprehensive Assessment of Ultraviolet-Radiation-Induced Mutations in Flammulina filiformis Using Whole-Genome Resequencing
Source: J Fungi (Basel). 2024 Mar 20;10(3):228. doi: 10.3390/jof10030228 (PMC10971301; doi:10.3390/jof10030228)
Supplement: Supplementary file 1 [file jof-10-00228-s001.zip › Supplementary Material S8/KEGG annotation/out/64381550635650.os/KO/out_map/map04914.html]

KEGG PATHWAY: Progesterone-mediated oocyte maturation - Reference pathway


|  |  |
| --- | --- |
| **Progesterone-mediated oocyte maturation - Reference pathway** |  |

[
Pathway menu
| Organism menu
| Pathway entry
| Show description
| User data mapping
]

Xenopus oocytes are naturally arrested at G2 of meiosis I. Exposure to either insulin/IGF-1 or the steroid hormone progesterone breaks this arrest and induces resumption of the two meiotic division cycles and maturation of the oocyte into a mature, fertilizable egg. This process is termed oocyte maturation. The transition is accompanied by an increase in maturation promoting factor (MPF or Cdc2/cyclin B) which precedes germinal vesicle breakdown (GVBD). Most reports point towards the Mos-MEK1-ERK2 pathway [where ERK is an extracellular signal-related protein kinase, MEK is a MAPK/ERK kinase and Mos is a p42(MAPK) activator] and the polo-like kinase/CDC25 pathway as responsible for the activation of MPF in meiosis, most likely triggered by a decrease in cAMP.

|  |  |
| --- | --- |
| Reference pathway | 100% |
